# Etiological factors in second mandibular molar impaction

**DOI:** 10.4317/jced.51382

**Published:** 2014-04-01

**Authors:** Michele Cassetta, Federica Altieri, Sabrina Calasso

**Affiliations:** 1 DDS PhD, Assistant Professor. Department of Oral and Maxillofacial Sciences, School of Dentistry, “Sapienza” University of Rome, Italy; 2 DDS, Assistant Researcher. Department of Oral and Maxillofacial Sciences, School of Dentistry, “Sapienza” University of Rome, Italy

## Abstract

Objectives: The impaction of the second mandibular molar (MM2) has recently become more prevalent. Several etiological hypothesis have been proposed to investigate the association between skeletal features and impaction of MM2. The aims of this study were to analyze the skeletal features in patients with MM2 impaction and the association between arrested eruption of MM2 and the presence of the third mandibular molar (MM3). Study Design: In this retrospective study 48 subjects from 3,530 Caucasian orthodontic patients with MM2 impaction were included in a study group (SG) and compared to a control group (CG) of 200 subjects without MM2 impaction. Panoramic radiographs evaluated the presence or absence of the MM3 germ. Cephalometric analysis was performed to evaluate linear and angular skeletal values. For the statistical analysis, descriptive statistics, Student’s t-test, χ2 test and odds ratio (OR) were used. Results: The paired comparisons between SG and CG showed in cephalometric analysis both a reduced mandibular gonial angle (ArGoMe) and lowered Jarabak’s polygon value with a statistically significant difference (P≤ 0.05). MM3 was statistically significant associated (P≤ 0.05) with MM2 impaction but it is not a risk factor (OR 0.817). Conclusions: Subjects with MM2 impaction show a vertical condylar growth direction. MM3 is not a risk factor for MM2 impaction.

** Key words:**Impacted mandibular second molar, skeletal features, orthodontic.

## Introduction

The impaction of the second mandibular molar (MM2), although a relatively rare occurrence, has recently become more prevalent (1-12). Concerning the MM2 impaction several etiological hypothesis have been proposed. An association between impaction of MM2 and the occurrence of crowding in the lower jaw was hypothesized. It was also supposed that a decreasing rate of extraction of the lower first permanent molar could be responsible for the increasing trend toward MM2 impaction (2). Some authors evaluating the different inclination of MM2, stated that the lack of space was the cause of retention of MM2 in the mesio and disto-angular positions, whereas local factors ,for example the ankylosis, were the reasons of retention in a vertical position (7). Other authors observed that MM2 impaction in patients undergoing non-extraction via E-space preservation with a passive lingual arch was 10 to 20 times more prevalent than that observed in general population and they suggested that any biomechanical approach that prevents mesialization of the first mandibular molar could produce similar results (13,14).

In a recent study some of the possible etiological factors relating to the MM2 impaction, like crowding, a higher angle of inclination of MM2, and a smaller distance between first mandibular permanent molar (MM1) and anterior margin of mandibular ramus were investigated (6). Few studies linked the facial pattern and the skeletal relationship to the second mandibular molar impaction (13,15,16).

The aim of the present study was to determine the skeletal features of subjects with this eruptive disorder and to investigate the presence of the third mandibular molar (MM3) as a risk factor in MM2 impaction.

The hypothesis of this study was that subjects with MM2 impaction show a vertical condylar growth direction; moreover, the germ of MM3 is a risk factor of MM2 impaction.

## Material and Methods

In this retrospective study MM2 was considered impacted if its complete eruption to occlusal height was prevented by an abnormal contact with another tooth in the same arch or when it remained unerupted beyond the time when it should normally erupt (1,2,7,9).

In this study an analysis of the pretreatment records of 3.530 Caucasian patients was performed. Subjects with at least one impaction of a second mandibular molar were selected for the study. The impaction diagnosis and the impaction site were determined on the basis of clinical examinations and standardized panoramic radiographs were made at the time of two-third of MM2 root formation (T2). 48 subjects with MM2 impaction were included in a study group (SG) and compared to a control group (CG) of 200 subjects without MM2 impaction, randomly chosen from the 2,180 remaining records that satisfied the following criteria:

– Children older than 10 years;

– Patients with no systemic syndromes;

– The availability of a lateral radiograph, taken in a cephalostat, obtained at the time of one third of MM2 root formation (T1);

– The availability of a panoramic radiograph with a magnification rate of 1:1 made at the time of two thirds of MM2 root formation (T2);

– All radiograms of sufficiently high quality.

All patients with MM2 impaction also fulfilled these criteria.

The cephalometric analysis was performed by tracing radiographic landmarks on acetate overlays and measuring linear and angular values. The skeletal class, the facial type and the direction of growth were evaluated.

The tracings were made on ultrathin 0.003 inch transparent acetate sheets using a Pentel 0.3 mm lead pencil. All the cephalometric radiographs were evaluated on a masked, illuminated viewbox in a room with reduced ligh-ting, and measured manually.

Seventeen reference points were selected ( Table 1). The cephalometric sagittal, vertical, dental ,and growth parameters evaluated in this study to estimate the craniofacial skeletal relationship are shown in  Table 2.

**Table 1:**
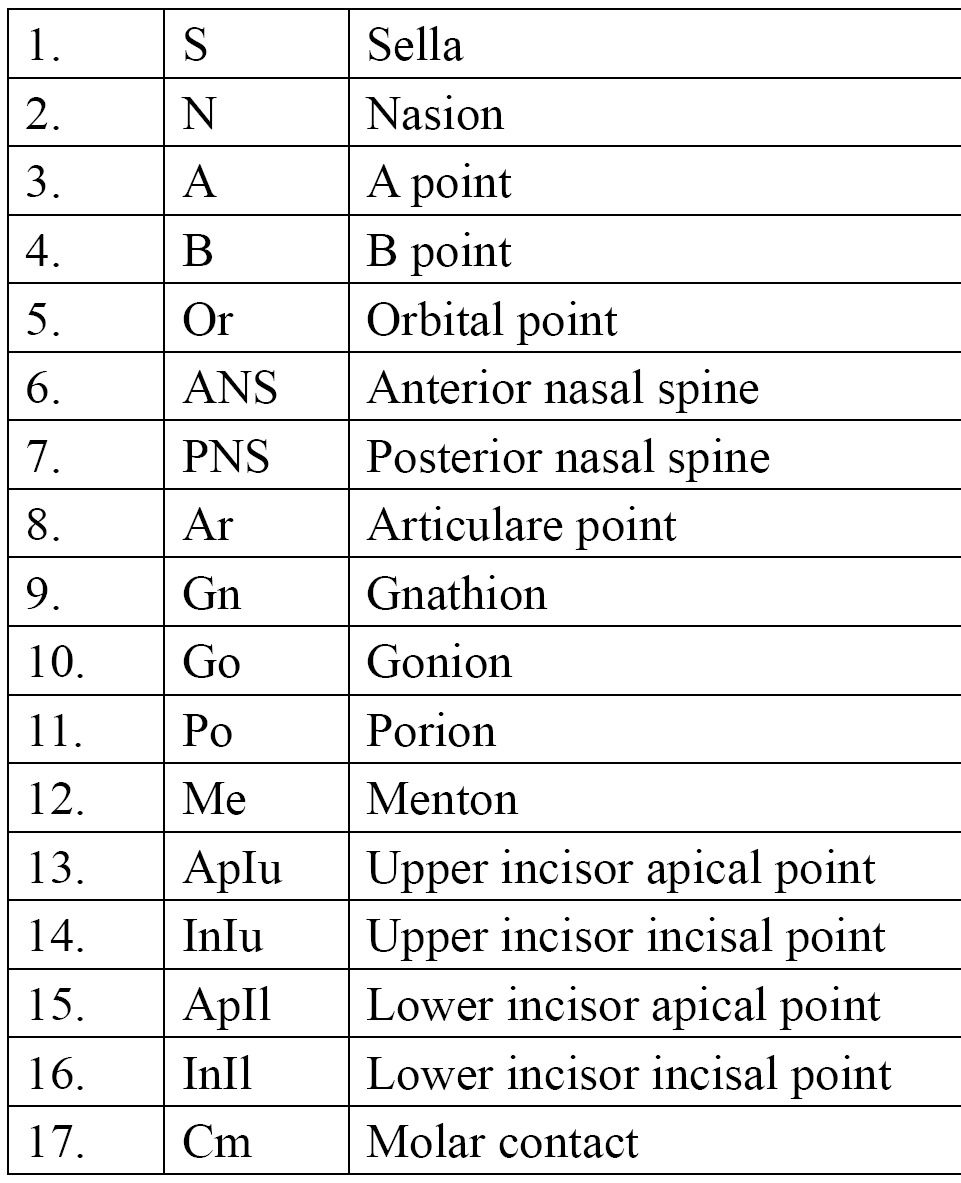
List of cephalometric points.

**Table 2:**
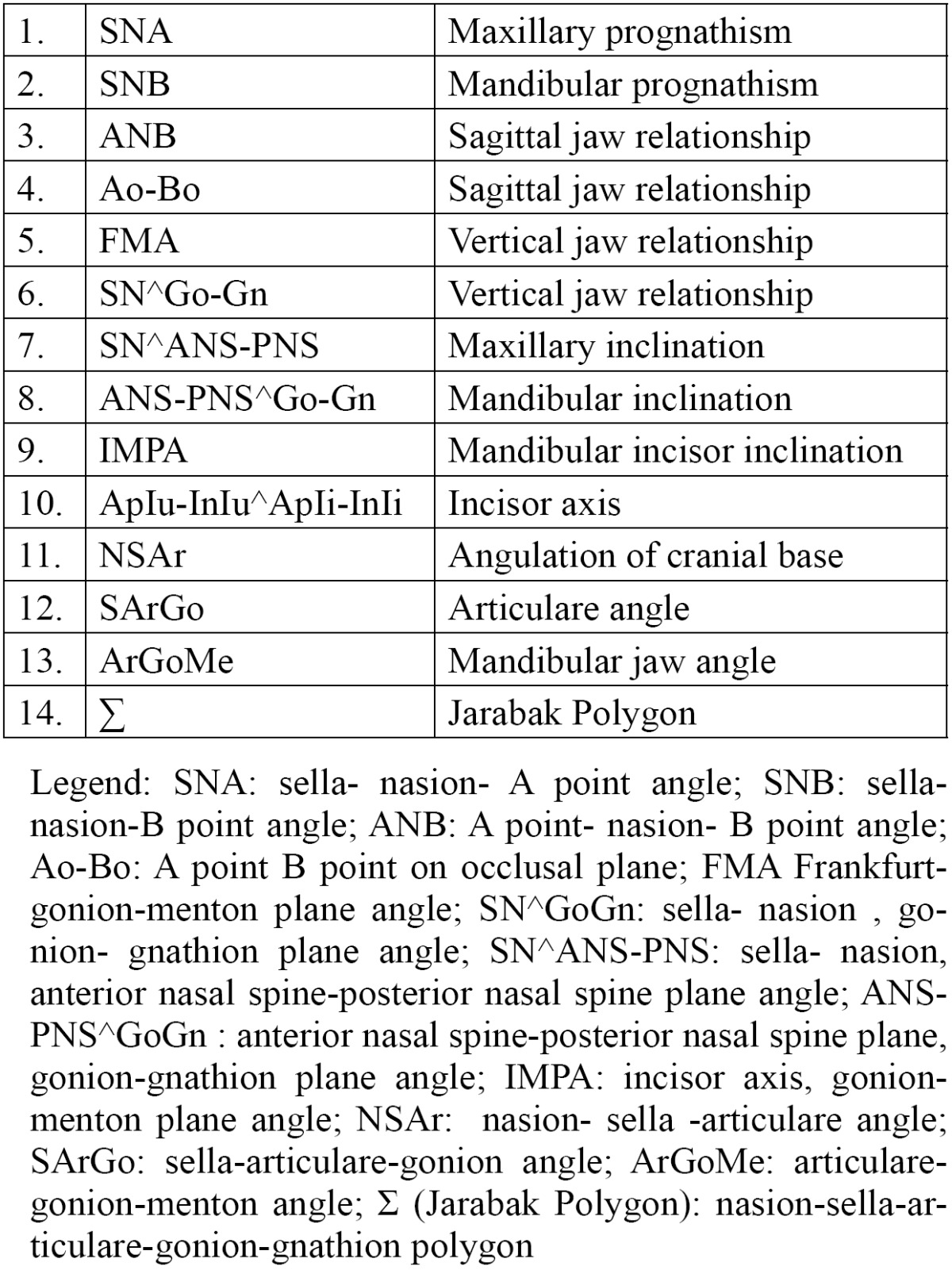
List of cephalometric variables.

**Figure 1:**
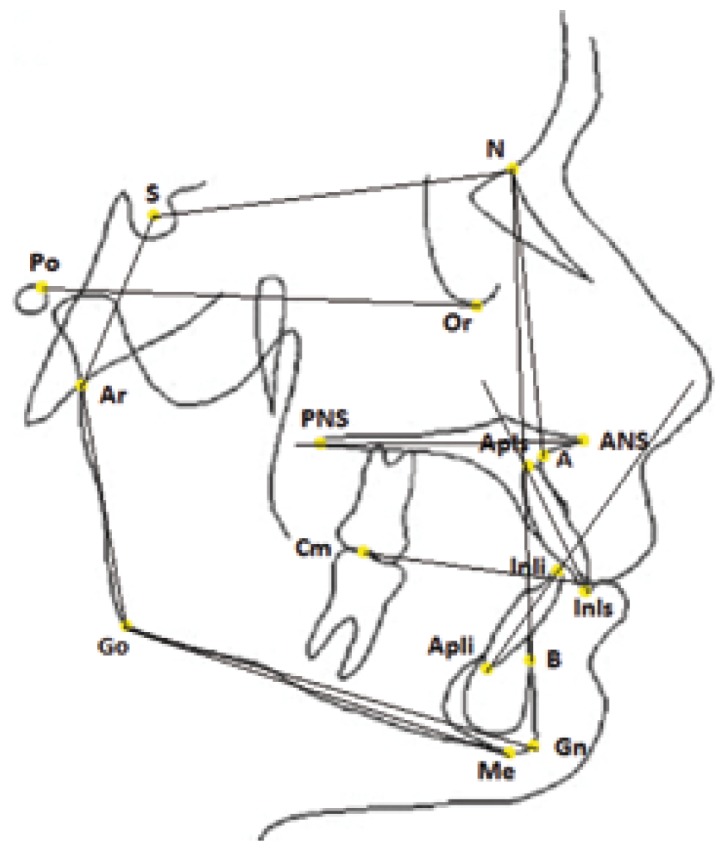
Diagram of contours and cephalometric landmarks used in the cephalometric evaluation of a lateral cephalogram.

Thirteen angular and one linear measurements were drawn ( Table 2, Fig. 1). The registration of all variables was performed by an expert orthodontist and the reproducibility of cephalometric measurements was assessed by reexamining the lateral cephalometric radiographs of 25 randomly selected patients 2 weeks after the first examination, by a single operator. Reproducibility was 100% for all variables except for FMA (98%) and SN^GoGn (96%).

The presence of a MM3 germ was determined on the panoramic radiograph performed at T2.

The local ethical committee was informed about the study protocol. The Helsinki Declaration was read and the guidelines followed in the present investigation.

Statistical descriptive analysis was performed and data were analyzed using SPSS software (Statistical Package for the Social Sciences, IBM Corporation, New York, NY).

The statistical analysis was conducted at individual level in the assessment of the distribution of subjects with MM2 impaction according to age and sex and also for cephalometric values. Descriptive statistics, consisting of mean, minimum-maximum and standard deviation, were calculated for each group; in addition the reproducibility and each cephalometric variable were tested by Student’s t-test. The levels of significance were set at *P* value ≤ 0.05.

Regarding the presence of MM3 the statistical analysis was conducted at teeth level. The analysis of association between MM2 impaction and the presence of MM3 was performed using the χ2 test, which was assumed to be significant when the p-value was not greater than 0.05 (*P*≤ 0.05). The odds ratio was used to assess whether the third mandibular molar is a risk factor of MM2 impaction.

## Results

Dental records of 3,530 subjects (1,872 females and 1,658 males; sex ratio: 4:3; mean age 14.84 ) were examined. From the study sample, 48 subjects were found with 68 impacted MM2 (27 male and 21 female; sex ratio 4:3). Bilateral impaction was seen in 20 patients, corresponding to 41.7%. Among the 28 patients with unilateral impaction, 21 impactions were seen on the right side (43.7%) and 7 on the left side (14.6%).

Cephalometric analysis results of SG and CG are shown in  Table 3. The sagittal jaw relationships (ANB, Ao-Bo) in each group were of skeletal Class I.

**Table 3:**
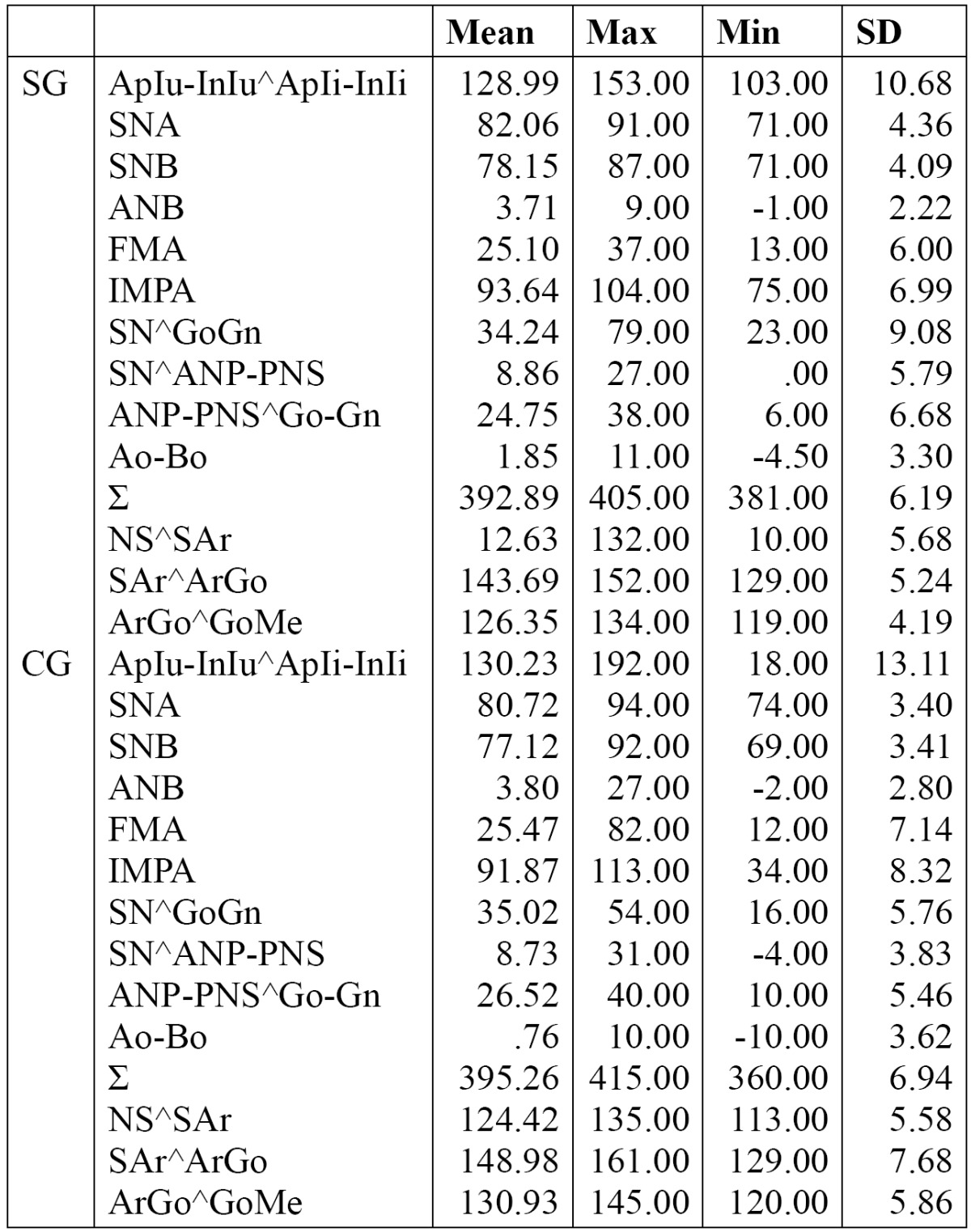
SG and CG skeletal features (descriptive statistical analysis).

The vertical analysis (FMA, SN^GoGn) showed normal values in each group studied.

However, FMA value was found smaller in SG than the CG but without a statistically significant difference.

The mandibular jaw angle (ArGoMe) in the SG was 4.6 degrees smaller and differed significantly from the CG (*P*<0.05); a statistically significant difference (*P* <0.05 ) for SNA and ∑ values was also determined ( Table 4).

**Table 4:**
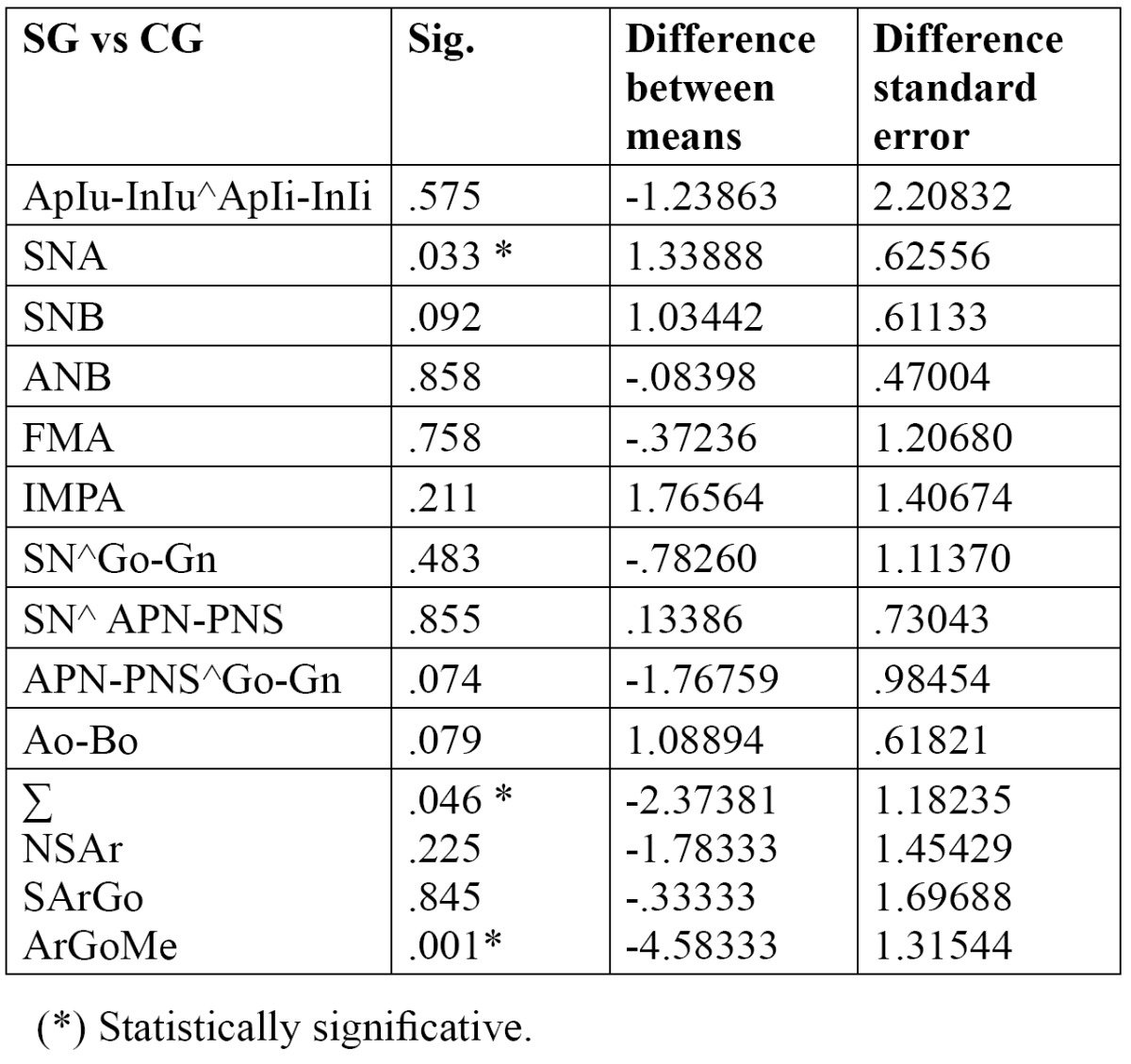
.T-test regarding the skeletal features between SG and CG ( *P*≤0.05).

There were no significant differences regarding the other cephalometric variables studied ( Table 4).

χ2 determined a statistically significant association between MM2 impaction and MM3 (*P*≤ 0.05) whereas the odds ratio was less than 1 ( OR 0.817) showing that MM3 is not a risk factor of MM2 impaction. This result did not confirm the study hypothesis.

## Discussion

To identify any association between craniofacial morphology and the occurrence of an arrested eruption of MM2, such as occurs in other disorder of eruption (15), sagittal analysis was performed in order to examine the antero-posterior relationships of the two jaws, and this showed normal ANB and Ao-Bo values in both groups investigated ( Table 3, Table 4). This result disagrees with data reported by other authors who found a statistically significant difference between the two samples examined (study and control group), with a tendency to skeletal Class II in patients with MM2 impaction (15).

Vertical analysis in each group showed normal FMA and SN ^ GoGn values (high percentage of normodivergent subjects) in agreement with other studies (13,15).

When the direction of growth was considered however, the results of this study revealed a slightly reduced Jarabak’s polygon value (Σ = 392.89 °) with a statistically significant lower ArGoMe value in the SG. This could suggest a vertically directed condylar growth, and showed that a link exists between craniofacial morphology and the occurrence of an arrested eruption of MM2 (15).

This finding was similar to that stated by a previous author with regard to the MM3, the eruption being directly proportional to the amount of space available, measured on a lateral radiograph, between MM2 and mandibular ramus and it is correlated to the condylar growth direction (17). When the condylar growth direction is vertical, the third molar may stay impacted, for the reduced resorption of the mandibular ramus anterior margin.

Therefore, based on previous and current study’s findings, it can be suggested that, in case of MM2 impaction, a vertical condylar growth direction could determine the reduced back molar space (17). This would explain the reduction of space between the distal marginal ridge of the first lower molar and the anterior border of the ramus that was found in a previous study (6).

Concerning the germ of MM3 as a risk factor of MM2 impaction, previous studies found that the third molar adjacent to an impacted second molar is seldom absent (2,4,7,15). A study observed the presence of third man-dibular molar in 85 % of the sample analyzed (2). Another study pointed out that mandibular third molars were developing in all but one case of MM2 impaction (4). Other authors described the presence of third molar adja-cent to the second molar in all but five of 88 patients with MM2 impaction (7). As mentioned by an earlier study, all patients with MM2 impactions had the germ of the third permanent molar, which is normally only seen in 63.4 - 77.5% of cases (15). In the present study, mandibular third molars were seen developing in the panoramic radiographs in all MM2 impaction cases.

Despite this frequent presence of MM3, some authors found no statistical significance between third molar presence and second molar impaction (13). Likewise, in the present study the statistical analysis did not indicate this condition as a risk factor of MM2 impaction (OR 0.817).

## Conclusions

The study results are.

• Skeletal features of MM2 impacted subjects suggest that a vertical condylar growth direction could be hypothesized.

• The third mandibular molar MM3 is not a risk factor for MM2 impaction.
